# Understanding negative feedback and classical conditioning of autonomic responses in physiological regulation

**DOI:** 10.3389/fphys.2026.1912305

**Published:** 2026-09-08

**Authors:** Douglas S. Ramsay, Stephen C. Woods

**Affiliations:** 1 Department of Oral Health Sciences, University of Washington, Seattle, WA, United States; 2 Department of Psychiatry and Behavioral Neuroscience, University of Cincinnati, Cincinnati, OH, United States

**Keywords:** anticipatory responding, feedforward control, homeostasis, Pavlovian conditioning, thermoregulation

## Abstract

Physiological regulation is achieved via a complex and highly integrated network of innate autonomic and somatic reflexes combined with learned responses acquired through experience. Historically, scholars investigating homeostatic regulation focused on the intricate innate reflexes, with little or no consideration of the key role of experience and learning. Our goal in this perspective paper is to provide increased clarity as well as a more nuanced understanding of the roles of negative feedback and feedforward mechanisms in thermoregulation with relevance to the broader field of physiological regulation. While physiological regulation requires innate regulatory reflexes and negative feedback control, we contend that learning also plays a key role when animals are confronted with common or recurring regulatory challenges. Animals readily acquire knowledge as to when regulatory disturbances are likely to occur and, accordingly, can learn to make anticipatory responses that minimize or eliminate completely the expected disturbance. We focus on how regulatory responses learned via classical conditioning enable more rapid and more efficient regulation than would result from negative feedback alone. Learning builds upon the foundation provided by innate regulatory reflexes and negative feedback and thereby improves the strength and timing of regulatory responses. We argue that a more inclusive approach to understanding physiological regulation, one recognizing the important roles played by both innate and learned reflexes to achieve regulation, will yield more innovative and productive research.

## Introduction

Thermoregulation is commonly viewed as an exemplar for biological regulation in general, and several recent reviews discuss the complex circuitry of the reflexes that underlie it (Notley et al., 2023; Mitchell et al., 2025a, 2025b). While scholarly, those reviews focus on negative feedback and feedforward mechanisms without adequately addressing the importance of learning in physiological regulation, including thermoregulation. In contrast to the broad recognition of learning’s role in behavioral thermoregulation^1^ (e.g., instrumentally learned behaviors such as adjusting a thermostat, selecting appropriate clothing, and/or moving to a preferred ambient temperature), there has been less appreciation that autonomic responses can also be learned and consequently elicited in anticipation of an expected thermal disturbance (Ramsay and Woods, 2014, 2016). Our goal is to describe classically conditioned autonomic responses and their importance to physiological regulation.

## Background

Negative feedback is a reactive regulatory mechanism whereby innate sensor-effector loops detect a physical stimulus (at the sensor) that then elicits a response (effector activity) that results in a change of the physical stimulus being detected. This strategy is reactive because the regulatory disturbance must already be present to activate the sensor-effector loop before the effector response(s) can be initiated (Ramsay and Woods, 2016). In contrast, activating regulatory responses in anticipation of an impending disturbance is a proactive regulatory control strategy that has advantages relative to a reactive approach.

The term ‘feedforward’ engages the concept of anticipatory control. It reflects the application of engineering concepts that use a correlate of an impending regulatory disturbance to activate effector responses prior to the disturbance, an approach recognized as being relevant to physiology (Houk, 1988). An example of innate hard-wired feedforward control is when the biochemical consequences of exercise provide a feedforward signal that activates thermo-effectors prior to the occurrence of an exercise-induced increase in body temperature (Notley et al., 2023). Learned responses made in anticipation of a disturbance were recognized as being relevant to physiological regulation, and as was customary at the time, were also described using the term feedforward (Siegel et al., 1987; Ramsay et al., 1996). To better distinguish anticipatory control resulting from learned responses from responses elicited via innate hard-wired mechanisms, we now prefer using the term ‘learned anticipatory responses’ although feedforward is still commonly used in the context of learning. For example, when discussing the complexity of homeostasis and physiological regulation, Billman (2020, pp. 7-8) states that “learning and experience provide the information necessary for feedforward control” such that the nervous system can use sensory input to elicit behavioral and physiological responses “*before* changes in the regulated variable have actually occurred.”

Siegel (2008, p. 251) asserts that the laws of learning must be understood to comprehend “the crucial contribution of learning to homeostatic regulation.” As explained by Dworkin and Dworkin (1999), a substantial literature documents that classical conditioning can directly modify autonomic reflexes that control visceral regulatory function, stating that the “classical conditioning process is always part of the closed-loop negative feedback mechanism of the unconditioned physiological reflex” (p. 160). Accordingly, the unconditional response (UR) refers to the effector activation elicited via negative feedback, and as learning occurs with successive associations between the conditional stimulus (CS) and negative feedback’s unconditional stimulus (US) - UR physiological reflex, the CS comes to elicit a conditional response (CR) that “augments the effect of the unconditioned response” (Dworkin and Dworkin, 1999, p. 160). In the context of applying classical conditioning to autonomic homeostatic regulation, the augmentation occurs because the learned effector response (i.e., the CR) and the negative feedback effector response (i.e., the UR) are alike in terms of effector mechanism and the direction of effector action, and because the magnitude of each of their effects is summative. The combined actions of the CR and UR increase the strength and improve the timing of the overall regulatory response of the nervous system (for additional details see, Dworkin, 1993, 2007; Dworkin and Dworkin, 1995; Ramsay and Woods, 1997).

**Figure 1:**
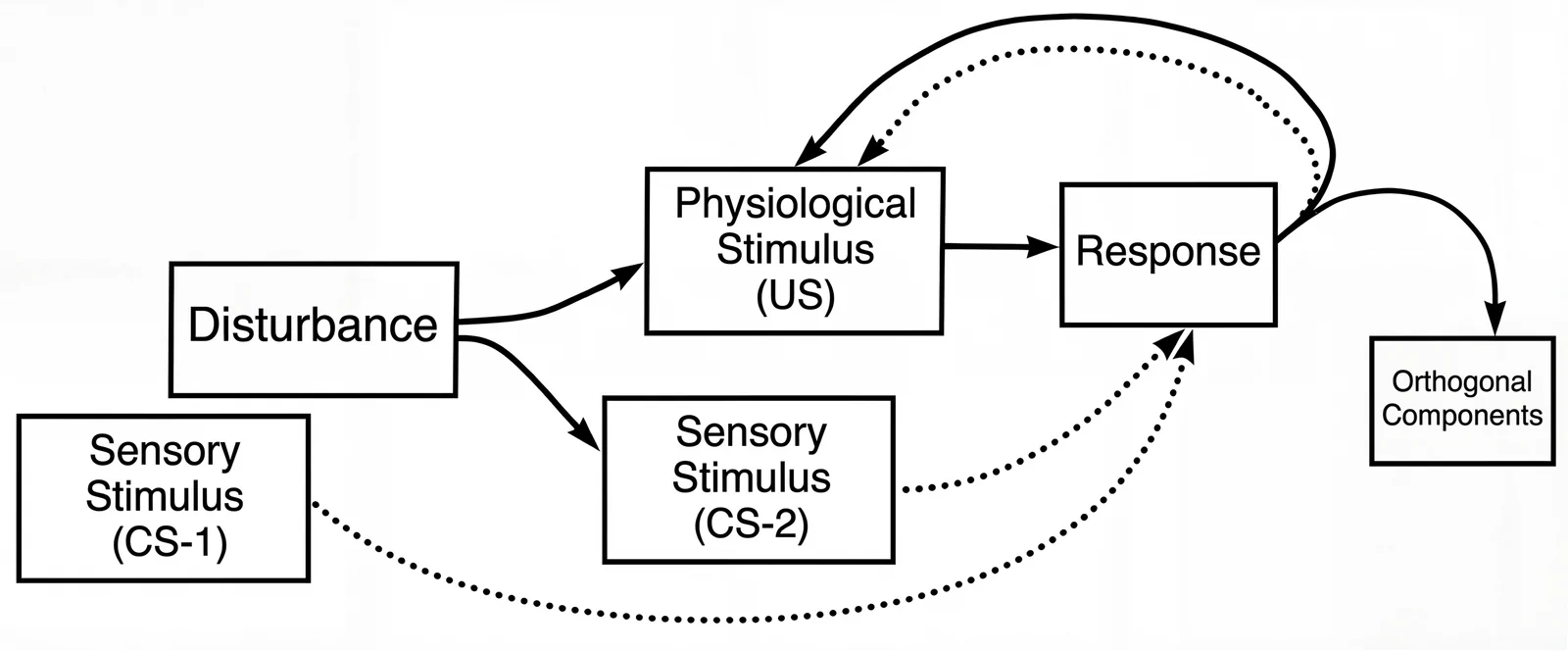
This diagram, adapted from (Dworkin, 1993, 2007; Dworkin and Dworkin, 1995, 1999), illustrates how the learned (classically conditioned) reflex (dotted path) interacts with the unlearned innate negative feedback loop (solid path) to accomplish physiological regulation. A critical point for the current discussion, (also made by Dworkin, 2007; Dworkin and Dworkin, 1995, 1999), is that the CNS mediated “Response” shown in the diagram represents the combined actions of both the unlearned negative feedback responses (URs) and learned conditional responses (CRs). The disturbance refers to the event that results in the physiological perturbation/unconditional stimulus that triggers the negative feedback regulatory response. The disturbance can also be associated with intero- and exteroceptive sensory components that anticipate the physiological stimulus such as the injection procedure (including sight and sensation) for a substance, the smell or taste of an ingested substance, or the time of day when the disturbance occurs. Thus, learning can occur with a sensory stimulus (e.g., CS-1) that predicts the disturbance and its resulting US or with a sensory stimulus (e.g., CS-2) that occurs with the disturbance and predicts the US. With experience, these sensory stimuli can serve as conditional stimuli (CSs) that elicit CRs, which participate in physiological regulation. [Non-regulatory components of the response that do not impact the variable under investigation are shown in the diagram as “Orthogonal Components” and described by Dworkin (1993). The diagram (used by permission from Dworkin, 1993) was modified by: 1) adding CS-1 that does not originate from the disturbance and has a dotted path to the response, and 2) labelling the physiological stimulus as the US.]. (Dworkin, (1993). Learning and Physiological Regulation (Chicago: University of Chicago Press)).

The underappreciated, and too often unacknowledged, elephant in the room of physiological regulation is that regulation is commonly accomplished using both learning and negative feedback (Dworkin, 1993; Siegel, 2008). Together, learning and negative feedback produce an augmented overall response strength that typically opposes a regulatory perturbation to homeostasis. The strength of the overall response results from the summation of conditional and unconditional responses (**Figure 1**). The anticipatory contribution of learned responses also improves the timing of regulatory responses (Ramsay and Woods, 2016). Dworkin and Dworkin (1999) explain that classically conditioned learned responses are an additional regulatory component that is built upon the foundation of negative feedback’s US-UR physiological reflex. Thus, classically conditioned regulatory responses would not exist without the foundation provided by the reflexive connection between the US and the UR inherent to negative feedback. Moreover, URs activated by negative feedback are important to prevent the CR from extinguishing (Dworkin and Dworkin, 1999). And so, as learning occurs, the nervous system’s regulatory response often consists of an integrated combination of learned and unlearned responses that *together* impact the physiological stimulus (i.e., the US) that triggers the innate reflex (**Figure 1**).

Both Siegel (2024, p. ix) and Woods trace their insights about physiological regulation and learning to their research on the regulation of blood glucose levels and their disturbance by insulin administration (Woods et al., 1969; Woods and Porte, 1974; Woods and Kulkosky, 1976). Woods proceeded to study eating and body weight regulation as well as the development of drug tolerance, and he eventually realized that analogous learning mechanisms were operating in both phenomena (Woods, 1991). At the time, the widely accepted view was that disturbances of homeostatically regulated^2^ physiological systems were corrected via negative feedback regulatory responses (Langley, 1973). Eventually, classically conditioned learned responses began to be recognized as an important component of physiological regulation (Dworkin, 1993; Ramsay et al., 1996; Ramsay and Woods, 1997; Siegel and Allen, 1998; Domjan, 2005; Woods and Ramsay, 2007; Siegel, 2008; Berntson et al., 2019).

A concept in classical conditioning relevant to this discussion is the distinction between heterotopic and homotopic conditional reflexes (Dworkin, 1993, pp. 29, 79-84; Dworkin, 2007; Siegel, 2008). Convincing experimental demonstrations of classical conditioning typically use heterotopic learning where the CS is distinctly different from the sensory characteristics of the US (e.g., Pavlov’s classic experiment where an auditory CS signaled the upcoming intraoral delivery of food). In contrast, homotopic conditional reflexes use a CS and a US that have similar sensory characteristics (e.g., both tastes) with the CS often being a less intense version of the US and with the CS always preceding the US. Having a CS with similar sensory characteristics as the US is believed to facilitate conditioning. Indeed, homotopic conditioning, where the CS is a similar but less intense version of an impending US, has been suggested to be more common and ubiquitous than heterotopic conditioning (Dworkin, 1993, 2007; Siegel, 2008).

A procedural description of a classical conditioning experiment pertinent to the current discussion is one in which a subject is given repeated challenges that disturb a regulatory system. The disturbance of the regulatory system leads to an US that reflexively elicits an UR within a negative feedback loop. In homeostatic regulation, the UR opposes or arrests the US. Within a heterotopic experiment, an initially neutral cue that has no impact on the regulatory system being measured, is reliably presented prior to the US, and over repeated trials the cue becomes associated with the US that reflexively activates the UR. Once learned, this initially neutral cue becomes a CS, and its presentation elicits a CR (Ramsay and Woods, 1997). This process is likely familiar to many. Domjan (2005, p. 179) provides a deeper understanding by explaining that the value of this learning is not to demonstrate that a once-neutral cue can now, as a CS, elicit a CR. Rather, its functional significance pertains to how the CR impacts the US, considering that the US is “the more biologically relevant stimulus.” Domjan (2005, p. 179) also makes the critical point that CSs are usually not initially neutral or arbitrary relative to the US but rather have a “pre-existing relation to an unconditioned stimulus.” This kind of pre-existing relationship between the CS and the US can result in homotopic classical conditioning.

**Figure 2:**
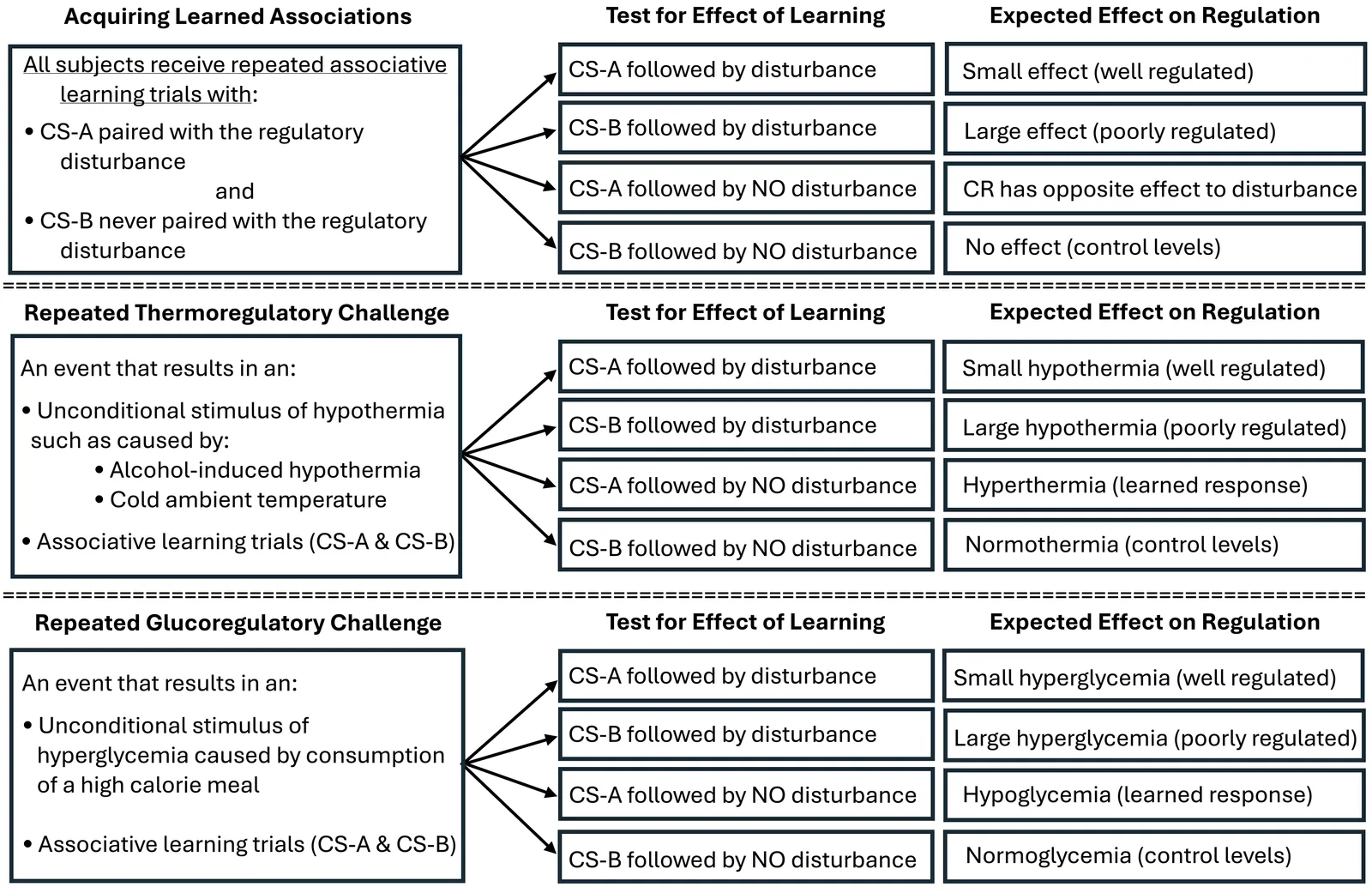
An experimental paradigm to evaluate the contribution of a classically conditioned response to physiological regulation. Subjects learn the predictive association between a stimulus and a pending regulatory disturbance. Subjects are then randomly assigned to one of four test conditions to determine if a conditional response (CR) impacts regulation. The top row describes the paradigm while the middle and bottom rows apply this approach to physiologically regulated systems.

The experiments discussed below are of two general types where subjects receive repeated regulatory disturbances within a heterotopic classical conditioning procedure and then are either: 1) given the event that causes the regulatory disturbance (i.e., a US that activates the negative feedback loop) with or without the CS, or 2) not given the event that causes the regulatory disturbance (i.e., the US does not occur to activate the negative feedback loop) with or without the CS. **Figure 2** describes this experimental paradigm and its expected results when investigating the role of learning in the physiological regulation of body temperature and blood glucose.

## Demonstrations of learned autonomic responses on physiological regulation

Early studies investigating the role of classical conditioning in the development of tolerance to the hypothermic effect of alcohol had a significant impact on the field (Lê et al., 1979; Mansfield and Cunningham, 1980; Crowell et al., 1981). These studies gave rats repeated administration of alcohol in association with distinct environmental cues. The initial regulatory disturbance of alcohol-induced hypothermia lessened over repeated alcohol administrations as tolerance developed. After substantial tolerance to alcohol-induced hypothermia occurred, rats were tested for evidence of learning. Tolerant rats that received alcohol in the absence of the CS previously associated with alcohol exhibited a greater hypothermic disturbance (i.e., they lost tolerance), despite negative feedback being available to arrest the hypothermia. These studies also found that when tolerant rats were given the CS associated with receiving alcohol, but were administered placebo instead of alcohol, they became *hyper*thermic. This was because the CS elicited a CR that would have opposed the anticipated US (the hypothermic effect of alcohol) had the US occurred. Research on non-drug induced hypothermic regulatory challenges (e.g., brief cold-water immersion) also supports the role of learning in thermoregulation (Riccio et al., 1991; Kissinger and Riccio, 1995; Siegel, 2008).

In the case of blood glucose regulation, presenting a CS that signals an impending meal elicits cephalic insulin release prior to initiation of the meal (Steffens, 1976; Berthoud et al., 1980; Grill et al., 1984; Powley and Berthoud, 1985; Lorentzen et al., 1987; Langhans et al., 2023). During the meal, the CR of cephalic insulin in combination with the insulin released via negative feedback from the meal itself, further curbs the meal-induced increase in blood glucose, i.e., if the CR is not elicited due to omission of the CS and food is consumed, subjects can become hyperglycemic (Louis-Sylvestre, 1976; Teff and Engelman, 1996; Ahrén and Holst, 2001; D’Alessio et al., 2001). If the CS for a meal is presented and consumption of the meal is prevented, the elicited CR can result in *hypo*glycemia (Permutt et al., 1977; Hofeldt, 1989). Reviews that include the human literature are available (Stockhorst et al., 2011; Teff, 2011; Wiedemann et al., 2020; Skvortsova et al., 2021).

In the case of drugs, classically conditioned regulatory responses are also learned over repeated drug exposures and play an important role in drug tolerance development (Ramsay and Woods, 1997; Siegel, 1976; Siegel et al., 2000). A compelling example involves the role of the learned response in reducing mortality from opioid overdose (Siegel et al., 1982). Rats received a series of escalating doses of heroin that were always administered in association with a distinct environment plus saline injections that were always administered in a distinctly different environment that was never associated with heroin. To evaluate the role of classical conditioning on regulatory responses that develop over repeated heroin administrations, a large dose of opioid was then administered on a test day. A control group that had the same exposure to the different environments, but no history of opioid exposures, had significant mortality (96.4% died) when administered the heroin. Half of the rats that had received the prior heroin injections received the large test-day heroin dose in the environment that was always associated with heroin, and the other half received it in the environment that was never associated with heroin. Compared to the heroin-naïve control rats, the rats receiving heroin in the CS environment associated with heroin had less mortality (32.4% died). In contrast, the rats with the identical heroin exposure history had a significant two-fold larger mortality (64.3% died) when heroin was administered in the CS environment that was not associated with heroin. Thus, death from opioid overdose was reduced when the respiratory depression effects of heroin were opposed by the CR that was elicited by the CS of the heroin-associated environment. This phenomenon is also believed to explain some instances of human overdose (Siegel, 2001, 2016, 2024).

Learning’s important role in physiological regulation is emphasized by the CR’s major impact on the US. After learned regulatory responses are established, the omission of a CS prior to a regulatory challenge results in the recurrence of a substantial regulatory disturbance due to the absence of the elicited CR. Direct evidence of CRs is experimentally demonstrated when the CS is presented in the absence of the US. As described in the examples above, lack of an elicited CR leaves a UR that is less able to arrest or oppose a challenge-induced US (e.g., alcohol-induced hypothermia, meal consumption-induced hyperglycemia, or opioid-induced respiratory depression). Importantly, this less effective regulation occurs despite the innate (i.e., unlearned) US-UR negative feedback reflexes being present to respond to these regulatory disturbances. Preemptive activation of learned responses makes regulation more effective at preventing (or minimizing) regulatory disturbances. Further, the summative effector action of the CR and UR is more effective for physiological regulation than the UR acting alone.

The experiments described above all used heterotopic classical conditioning where the CS was initially a neutral stimulus and independent of the regulatory challenge, which facilitates presentation or omission of the CS for experimental purposes. However, in the case of homotopic classical conditioning, an early sensory component of the regulatory challenge and the resulting onset of the regulatory disturbance can function as a CS (e.g., a smaller, less intense version of the US) by signaling an upcoming larger regulatory disturbance (i.e., the full US). Kim et al. (1999, p. 502) have described this kind of homoreflex CS as an “ineluctable part” of the US. Though methods for studying this type of conditioning are not well established, research on homotopic conditioning is likely to have important implications for understanding physiological regulation. For example, failures to demonstrate classical conditioning in a heterotopic paradigm may result from an unrecognized more salient homotopic CS being critical to eliciting learned anticipatory responses. Interoceptive drug onset cues (Dworkin, 2007; Siegel, 2008) as well as internal cues related to deciding to self-administer a drug (Weise-Kelly and Siegel, 2001), or to initiate a meal, or to leave a relatively warm muskrat lodge and enter near freezing water (MacArthur, 1979), are examples that can all trigger anticipatory learned responses that prepare the individual to exhibit better regulation during the upcoming regulatory challenge. For further discussion of homotopic conditioning, see (Dworkin, 1993; Kim et al., 1999; Sokolowska et al., 2002; McDonald and Siegel, 2004; Dworkin, 2007; Siegel, 2008).

This brief perspective paper has presented influential literature that has shaped the current understanding of how classical conditioning of autonomic responses contributes to physiological regulation. It is worthwhile to apply this understanding to a compelling recent research investigation from the field of thermoregulation in which mice were classically conditioned with repeated cold exposures in a study that employed sophisticated engram-labelling technology, chemogenetics and optogenetics (Muñoz Zamora et al., 2025). In an analogous manner as described in **Figure 2**, mice learned to associate room temperature (21° C) exposures with Environment A and cold temperature (4° C) exposures with Environment B. During an initial cold exposure, thermoregulatory negative feedback control detects the US of cold and innately activates a UR of metabolic heat production. After repeated exposures, mice were tested for associative learning by presenting the CS of Environment B, except the ambient temperature was at 21° C instead of 4° C. Presenting the CS of Environment B elicited a CR of increased metabolic heat production and body temperature increased. Their study found that autonomic (e.g., brown fat thermogenesis) and behavioral regulatory responses are elicited by the CS regardless of environmental temperature, and that retrieval, or artificial reactivation, of a cold memory mediates the thermoregulatory responses. The findings supported their conjecture that “learned information could enable animals to anticipate thermal challenges on the basis of previous experience and allow them to respond in an energy efficient manner” (Muñoz Zamora et al., 2025, p. 942).

## Summary

The purpose this perspective article is advanced by further discussing the significance of the study by Muñoz Zamora et al. (2025). Had their experiment not been designed to assess associative learning, and instead had given repeated cold exposures to measure the thermoregulatory adaptations that develop, would the existence of the learned response and its contribution to the overall regulatory response have been recognized? In our view, this is unlikely considering that the UR and CR use the same effector mechanism. The increasing contribution of the CR of metabolic heat production would not be easily distinguished from the UR of negative feedback activated metabolic heat production. We suspect that the growth of the adaptive response would likely have been (mis)attributed to the UR of a negative feedback mechanism alone rather than a combined effect of the UR and CR. It was the experimental design that included the test trial for classical conditioning that made the CR apparent (**Figure 2**). The implications of their research and its future directions are discussed further by Toh and Rajbhandari (2025). The work by Muñoz Zamora et al. (2025) is an excellent example of the kind of research that we believe will advance the understanding of physiological regulation. We believe that learning needs to be embraced as one of the major processes that underlie physiological regulation along with negative feedback mechanisms. Doing so will lead to a better understanding of physiological regulation and stimulate insightful research.

## Data Availability

The original contributions presented in the study are included in the article/supplementary material. Further inquiries can be directed to the corresponding author.

## Footnotes

1 Learned instrumental behaviors are also important in physiological regulation. However, we do not address instrumentally acquired behaviors in thermoregulation because this review focuses on autonomic effector responses acquired via classical conditioning.

2 This article is written from the perspective of homeostasis, which remains the dominant framework for understanding physiological regulation. However, other more recent models (e.g., allostasis) have been proposed to explain regulatory phenomena that seem inconsistent with homeostasis (Ramsay and Woods, 2014). For example, Hammel (1989, 1990) suggested that positive feedback may explain some regulatory phenomena. Dworkin (1993, p. 84) speculates on the function of positive feedback in regulation. Kitano et al. (2004) posit that positive feedback may contribute to dysfunctional regulation in metabolic syndrome. An example of a positive feedback reflex is provided by Montano et al. (2000).
